# Supplementary material for: Repositioned Drugs for Chagas Disease Unveiled via Structure-Based Drug Repositioning
Source: Int J Mol Sci. 2020 Nov 20;21(22):8809. doi: 10.3390/ijms21228809 (PMC7699892; doi:10.3390/ijms21228809)
Supplement: Supplementary file 1 [file ijms-21-08809-s001.pdf]

Supplementary Materials: Supplementary Data: Repositioned Drugs for Chagas Disease Unveiled via Structure-Based Drug Repositioning

Melissa F. Adasme [1], Sarah Naomi Bolz [1], Lauren Adelmann [1], Sebastian Salentin [1], V. Joachim Haupt[1], Adriana Moreno-Rodríguez[2], Benjamín Nogueda-Torres[3], Verónica Castillo-Campos[3], Lilián Yepez-Mulia[4], José A. De Fuentes-Vicente [5], Gildardo Rivera [6], Michael Schroeder [1]*

Table S1. Cofactors excluded from the screen

Cofactor	HET ID
Flavin-Adenine Dinucleotide (FAD)	FAD
Magnesium Ion	MG
Dihydroflavine-Adenine Dinucleotide	FDA
Nicotinamide-Adenine-Dinucleotide (NAD)	NAD
Flavin Mononucleotide	FMN
Nicotinamide Adenine Dinucleotide Phosphate (NADP)	NAP
Heme	HEM
Dihydro-Nicotinamide-Adenine-Dinucleotide Phosphate (NADPH)	NDP

Table S2. Buffer or Solvent ligands excluded from the screen

Ligand	HET ID
Sodium Ion	NA
Di(hydroxyethyl)etherDI	PEG
Acetate ion	ACT
Sulfate ion	SO4
Triethylene Glycol	PGE
Hexane	HEX
Glycerol	GOL
Phosphate Ion	P04
HEPES	EPE
Maleic acid	MAE
2-(N-Morpholino)-Ethanesulfonic Acid	MES
Beta-Mercaptoethanol	BME
Ethylene Glycol	EDO
Dimethyl Sulfoxide	DMS
Cobalt Hexammine (III)	NCO
Dimethylarsinate	CAC
Chloride Ion	CL
1,4-Diethylene Dioxide	DIO
3-Fluorosialic Acid	FSI
2-Propanol	IPA
Ethanol	EOH

Table S3. PDB structures used in the screen that required a special preparation

Target	PDB ID	Ligand	Ligand Type	Preparation
FPPS, Homoallylic site	3IBA	Isopentyl Pyrophosphate	Substrate	alternate location
FPPS, Homoallylic site	3ICK	Isopentyl Pyrophosphate	Substrate	alternate location
FPPS, Homoallylic site	1YHM	Isopentyl Pyrophosphate	Substrate	alternate location
Lanosterol Demethylase	5AJR	VT-1161	Inhibitor	Split from Heme
Lanosterol Demethylase	3ZG3	UDD	Inhibitor	Split from Heme
Lanosterol Demethylase	3ZG2	UDO	Inhibitor	Split from Heme
Lanosterol Demethylase	3KSW	VNF	Inhibitor	Split from Heme
Lanosterol Demethylase	2WX2	Fluconazole	Inhibitor	Split from Heme
Lanosterol Demethylase	3KHM	Fluconazole	Inhibitor	Split from Heme
Lanosterol Demethylase	2WUZ	Fluconazole	Inhibitor	Split from Heme
Lanosterol Demethylase	3K1O	Posaconazole	Inhibitor	Split from Heme

Table S4. Parasitemia inhibition percentage over the time

Drug	**2 horas**		**4 horas**		**6 horas**		**6 horas**	
(100 mg/kg)	Ninoa	INC-5	Ninoa	INC-5	Ninoa	INC-5	Ninoa	INC-5
Ciprofloxacin	100	100	50	75	33.3	62.5	33.3	56.2
Naproxen	100	100	71	85.7	28.5	71.4	14.2	57.1
Folic acid	100	100	71	50	42.8	33.3	42.8	33.3
Nifurtimox	100	100	50	66.6	27.0	55.5	22.2	27.7
Negative control	100	100	100	100	100	100	100	100

Table S5. Pathways of all the targets

**Target**	**Pathway**	**Action**
DHFR	folate metabolism	reduce dihydrofolate
TS	Pyrimidine biosynthesis	methylate dUMP
Cruzipain	proteolysis	Cysteine Protease
Farnesyl Pyrophosphate Synthase	Ergosterol biosynthesis	isoprenoid synthesis
Glyceraldehyde 3-Phosphate Dehydrogenase	Energy Production	catalyzes sixth step of Glycolysis
Lanosterol 14-alpha Demethylase	Ergosterol biosynthesis	demethylates lanosterol
Trans-Sialidase	host immune response & invasion	cell surface enzyme
Trypanothione Reductase	trypanothione synthesis	reduction of trypanothione
B Cell Mitogen (Proline Racemase)	Host immune response	triggers host B cell polyclonal activation
Cyclic Nucleotide Specific Phosphodiesterase	cell proliferation & invasion	control cAMP levels
Dihydrolipoyl Dehydrogenase	energy metabolism	in enzyme complexes citric acid cycle
Dihydroorotate Dehydrogenase	pyrimidine biosynthesis	oxidation of oorate; reduction of fumarate
Glucose-6-Phosphate Dehydrogenase	pentose phosphate	generates NADPH
HGPRT	purine biosynthesis	convert guanine/hypoxanthine bases
Old Yellow Enzyme (Prostaglandin F2 Synthase)	drug metabolism	reduces various compounds
Pteridine Reductase	Pterin/folate biosynthesis	reduces pteridins
Ribose 5-Phosphate isomerase	pentose phosphate pathway	produce NADPH
Spermidine Synthase	trypanothione synthesis	generates trypanothione precursor
Squalene Synthase	Ergosterol biosynthesis	generates sterol precursor
Triosephosphate Isomerase	energy production	catalyzes fifth step in Glycolysis
UDP-Galactopyranose mutase	host immune response	cell surface sugar production
